# Evaluation of FTIR Spectroscopy as a diagnostic tool for lung cancer using sputum

**DOI:** 10.1186/1471-2407-10-640

**Published:** 2010-11-23

**Authors:** Paul D Lewis, Keir E Lewis, Robin Ghosal, Sion Bayliss, Amanda J Lloyd, John Wills, Ruth Godfrey, Philip Kloer, Luis AJ Mur

**Affiliations:** 1 School of Medicine, Swansea University, Swansea, SA2 8PP, UK; 2 Department of Respiratory Medicine, Prince Phillip Hospital, Llanelli, SA14 8LY, UK; 3 Institute of Biological, Environmental and Rural Sciences, Aberystwyth University, Aberystwyth, SY23 2AX, UK

## Abstract

**Background:**

Survival time for lung cancer is poor with over 90% of patients dying within five years of diagnosis primarily due to detection at late stage. The main objective of this study was to evaluate Fourier transform infrared spectroscopy (FTIR) as a high throughput and cost effective method for identifying biochemical changes in sputum as biomarkers for detection of lung cancer.

**Methods:**

Sputum was collected from 25 lung cancer patients in the Medlung observational study and 25 healthy controls. FTIR spectra were generated from sputum cell pellets using infrared wavenumbers within the 1800 to 950 cm^-1 ^"fingerprint" region.

**Results:**

A panel of 92 infrared wavenumbers had absorbances significantly different between cancer and normal sputum spectra and were associated with putative changes in protein, nucleic acid and glycogen levels in tumours. Five prominent significant wavenumbers at 964 cm^-1^, 1024 cm^-1^, 1411 cm^-1^, 1577 cm^-1 ^and 1656 cm^-1 ^separated cancer spectra from normal spectra into two distinct groups using multivariate analysis (group 1: 100% cancer cases; group 2: 92% normal cases). Principal components analysis revealed that these wavenumbers were also able to distinguish lung cancer patients who had previously been diagnosed with breast cancer. No patterns of spectra groupings were associated with inflammation or other diseases of the airways.

**Conclusions:**

Our results suggest that FTIR applied to sputum might have high sensitivity and specificity in diagnosing lung cancer with potential as a non-invasive, cost-effective and high-throughput method for screening.

**Trial Registration:**

ClinicalTrials.gov: NCT00899262

## Background

Lung cancer is the most common cancer in the world where 1.3 million deaths are recorded each year [1]. It is the second most common cancer in the UK and most common cause of cancer mortality with 34,500 deaths per annum [2]. Survival time is also poor with over 90% of patients dying within five years of diagnosis. Besides co-morbid conditions, poor survival rates primarily reflect the fact that over two thirds of patients are diagnosed at a stage that is currently not amenable to potentially curative treatment.

A number of reasons exist as to why so many lung cancers are diagnosed at late stage. The aetiology of lung cancer is well established with approximately 90% of tumours occurring in smokers [3]. Smoking is not just problematic in terms of the causation of lung cancer as common symptoms of lung cancer such as coughing, dyspnoea or haemoptysis are frequently caused by smoking itself so are dismissed by long term smokers as simply being a consequence of smoking. Current diagnostic methods include chest X-ray, computerized tomography (CT) and bronchoscopy but despite these methods improving the ability to detect lung cancer they remain less effective for early stage detection [4]. In reality, the detection of lung cancer at an early stage would require a national screening programme. Targets for screening would be those at high risk including people over the age of 60 with a history of smoking, those with a previous history of cancer, and patients with chronic obstructive pulmonary disease (COPD). However, a screening programme would require a technology that is sensitive to early stage lung cancer and cost effective with the ultimate aim to reduce mortality.

Recent randomized controlled trials have focused on the evaluation of low dose computerised tomography (LDCT) [5]. It is estimated that, although having acceptable cost effectiveness, LDCT would still be an expensive screening method [6]. Furthermore, there is debate as to the sensitivity of LDCT for use on asymptomatic high risk cases [7] and recent findings from the DANTE trial suggest that mortality reduction using LDCT as a screening tool might be smaller than anticipated [8].

Molecular markers for early detection of lung cancer hold much promise and possess a number of advantages over existing methods. Indeed, markers such as DNA methylation status of certain genes can be detected in biofluids such as blood and sputum from people with lung cancer [9] as well as broncho-alveolar lavage (BAL) fluid [10]. As opposed to LDCT, technologies that can detect molecular biomarkers involve no radiation exposure, are relatively inexpensive and high throughput so satisfying the need to have a screening process that is cost effective and rapid.

Fourier transform infrared spectroscopy (FTIR) is a non-invasive technology that can detect a change of functional group in molecules from tissue or cells. Such changes can be visualized using a spectrum of wavenumbers usually taken from the mid infrared range (4000 to 400 cm^-1^). FTIR has shown promise as a sensitive diagnostic tool to distinguish neoplastic from normal cells in cancers such as colon [11], prostate [12], breast [13], cervical [14], gastric [15], oral [16] and oesophageal [17]. In these and other studies, biochemical changes are often observed between tumour and normal cells within a wavenumber range known as the "fingerprint region" (encompassing 1800 to 950 cm^-1^).

There have been surprisingly few attempts to apply FTIR for diagnostic purposes in lung cancer tissue. This likely reflects the fact that access to lung tissue is difficult, highly invasive and most patients are diagnosed at a late stage. The few results that have emerged though are encouraging where FTIR wavenumbers in the fingerprint region were interpreted to suggest that elevated glycogen levels significantly discriminate both squamous cell carcinoma and adenocarcinoma tumours from normal tissue [18,19]. A related infrared technique, Raman spectroscopy, has also demonstrated success in distinguishing lung tumour from normal bronchial tissue [20] and has more recently been combined with a bronchoscope for potentially diagnosing lung cancer *ex vivo *[21]

FTIR has successfully been applied for diagnostic purposes on sputum from COPD patients [22] and for bacteria identification in cystic fibrosis [23]. However, despite success in detecting cancerous change in cells from pleural fluid [24], to our knowledge FTIR has never successfully been applied to sputum for lung cancer diagnosis. The aim of our study was to identify infrared wavenumbers that significantly discriminate cancer sputum from healthy control sputum and apply multivariate analysis to determine whether samples form sub-groups according to variation in wavenumber signal. This work lays the foundation for further studies to evaluate whether FTIR might be used as a cost effective, high throughput and non-invasive method for screening biochemical changes in sputum from lung cancer cases.

## Methods

### Study Subjects and Sputum Collection

This study had approval from the loco-regional ethical committee (05/WMW01/75). Spontaneous sputum was collected from 25 patients (mean age 66.5 ± 9.2 years; 15 males, 10 females), for the majority,(23/25) it was taken just prior to bronchoscopy for suspected lung cancer. Informed consent to provide a sputum sample was obtained from each patient at a previous clinical appointment. Lung cancer was subsequently confirmed through final clinical diagnosis) as part of the Medlung observational study (UKCRN ID 4682). Final histology was recorded where known (19 non-small cell lung cancer (NSCLC): 7 squamous cell carcinoma, 5 adenocarcinoma, 7 unknown histological subtype, 3 small cell carcinoma; 1 large cell carcinoma; 2 radiological diagnosis). The two patients who had a radiological diagnosis based on clinical presentation and radiological findings were too unwell for further investigation to determine histological subtype. Tumour location was also recorded for each of the 23 patients who underwent bronchoscopy: 11 normal (i.e. no tumour observed at bronchoscopy); 2 external compression of bronchus/trachea from the tumour/nodes; 4 abnormal mucosa; 6 tumour seen endobronchially. Based on CT scans all patients had a centrally located tumour. In total, 12 cancer cases (48%) showed no evidence of a tumour at bronchoscopy although random bronchial washings were still taken on all. Smoking status and pack years (pky) were recorded for each patient: 11 current smokers (median pky = 40); 11 ex smokers (median pky = 40); 3 never smokers. Sputum was also collected from 25 healthy donors (mean age 62.5 ± 11.1 years; 15 males, 10 females) consisting of staff members at Swansea University with no previous history of cancer or lung disease other than COPD or asthma. Smoking status and pack years recorded for controls were: 12 current smokers (median pky = 30); 5 ex smokers (median pky = 27); 8 never smokers.

### Sputum collection and processing

After collection, sputum samples were kept frozen at -80°C. Prior to processing, sputa were defrosted at room temperature for 12-24 hours. Sputum cells were isolated by breaking down the mucus with a solution consisting of 2.5 g Dithiothreitol (DTT) in 31 mL Cytolyt (Fluka Biochemika Sigma-Aldrich Chemie GmbH Switzerland) which digested mucus in the specimen. Samples then underwent centrifugation at 3000 rpm for 10 min and supernatant poured off leaving a cell pellet. An aliquot of cells was taken to create a second pellet that was subsequently formalin fixed and wax embedded prior to sectioning and staining with haemotoxylin and eosin (H&E). Residual pellets were freeze-dried over night, diluted in 200 μL of sterile distilled water, agitated for 5 min and split into 20 μL aliquots, which were frozen immediately in liquid nitrogen and stored at -80°C. To confirm samples were of bronchial origin, H&E stained sections were assessed by a consultant histopathologist for presence of bronchial epithelial cells.

### FTIR

A FTIR mid-infrared spectrometer instrument (Buker VERTEX 80/80 v, Ettlingen, Germany) was used to obtain spectra which comprised a KBr Beamsplitter, a diffuse reflectance absorbance scanning accessory equipped with a mercury-cadmium telluride (MCT) detector and a sampling compartment fitted with a horizontal attenuated total reflectance (HATR) sampling accessory. In this study, we used the MCT detector for reflectance IR measurements. To reduce noise, the MCT detector has to be operated at liquid nitrogen temperatures. Ninety-six well silicon plates (LNC Technology Ltd., Ystrad Mynach, Hengoed, UK) were cleaned in warm 0.5% SDS, rinsed with dH_2_O, soaked overnight in 5 M nitric acid, rinsed again with dH_2_O and air dried. Samples were applied randomly, in triplicate, across a plate, permitting possible variations within or between plates to be taken into account during analysis. Loaded sample plates were oven dried at 50°C for 30 min (Sanyo Gallenkamp plc., Loughborough, UK) to remove extraneous moisture prior to FTIR analysis. Prepared plates were allowed to cool and then inserted onto the motorized stage of the diffuse reflectance absorbance scanning accessory connected to the FTIR spectrometer. FTIR spectra were obtained in reflectance mode. Interference peaks in the mid-infrared region of spectra were collected to minimise noise from CO_2 _and H_2_O vapour. The sampling compartments and microscope stage were purged with dry CO_2 _free air produced from a Peak Scientific compressor (Peak Scientific Ltd. Paisley, UK). Data points were collected at a resolution of 2 cm^-1 ^from the 4000 to 600 cm^-1 ^wavenumber range. Each spectrum represented the average of 256 scans for improved signal to noise ratio. Sample absorbance spectra were calculated from the ratio of IS/IR, where IS was the intensity of the IR beam after it has been absorbed by the sample and IR was the intensity of the IR beam from the reference. The absorbance spectrum was calculated as: -log_10_(IS/IR).

### Data Processing and Analysis

All data processing, analysis and visualization were performed using the R statistical computing environment [25] using in-built algorithms and code developed by our group. We were interested in assessing change in the fingerprint spectral region so absorbance values between wavenumbers 950 and 1800 cm^-1 ^(442 data points) were pre-processed prior to further analysis. Raw spectra were were pre-processed using a simple two point linear subtraction baseline correction method. Two points, 900 and 1850 cm^-1 ^were selected outside the wavenumber region of interest that showed no variation across all samples. Spectra were then vector normalised. Second derivative spectra were then calculated after smoothing using the Savitzky-Golay algorithm with nine points. We applied the Shapiro-Wilk test to assess whether second derivative absorbance values for each number followed a normal distribution. As no wavenumber was found to be normally distributed the Mann-Whitney *U *test was used to determine wavenumbers that were significantly different (*P *= 0.05) between cancer and control spectra. Furthermore, due to multiple testing, a wavenumber p-value was only retained as significant if it remained so after applying Holm's sequential Bonferroni correction.

Two different multivariate analysis approaches were used to determine and visualize the pattern of similarities within and between cancer and normal spectra. Hierarchical cluster analysis (HCA) dendrograms were produced using the 'hcluster' function in the R 'amap' package with a correlation distance matrix and unweighted pair-group tree building method. Principal components analysis (PCA), via the R 'prcomp' package, allowed further visualization and interpretation of spectral groupings.

## Results

### Evaluation of sputum cell pellet FTIR spectra

**Figure 1:**
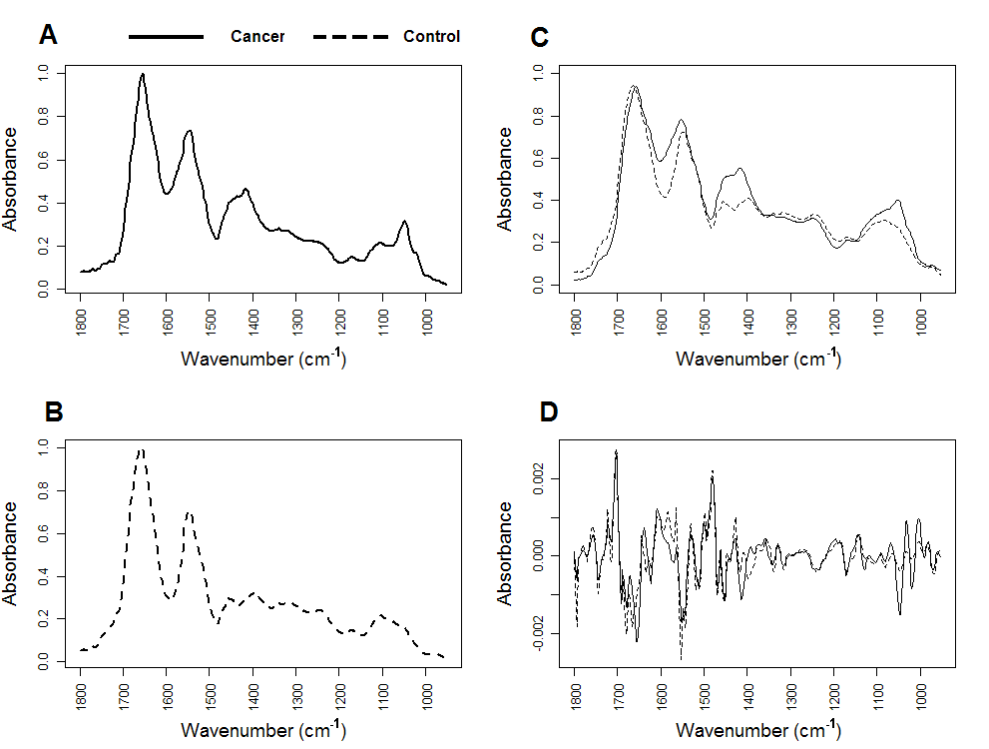
**Raw example FTIR spectra for cancer and normal sputum**. Raw example FTIR spectra between wavenumbers 950 cm^-1 ^and 1800 cm^-1 ^for (A) cancer sputum and (B) normal sputum. (C) Median raw spectra for cancer and normal sputa. (D) Second derivative spectra for cancer and normal sputa.

FTIR was used to generate absorbance spectra in the frequency region 950 to 1800 cm^-1 ^to establish potential metabolic differences in cells extracted from sputum between 25 lung cancer and 25 normal control samples. Representative spectra for cancer and control samples are shown in Figure 1 A-B. By repeating the procedure multiple times (and on different dates) we found that spectra generated for each sample were highly reproducible. Normality tests revealed that none of the individual wavenumber absorbance levels followed a normal distribution. Thus, for each wavenumber, median(rather than mean) absorbance levels were used for analysis.

Figure 1C shows median spectra for cancer and normal sputa highlighting key regions of wavenumber absorbance where differences exist between the two groups. The prominent absorbance bands observed in both cancer and normal spectra are characteristic of vibration modes assumed to represent functional groups in cellular molecules including proteins, carbohydrates and nucleic acids [26,27,24]. In all spectra, major peaks were observed for absorptions in the amide I and amide II regions at 1656 cm^-1 ^and 1577 cm^-1 ^respectively. Another broad region of peaks assumed to be associated with proteins was seen between 1400 cm^-1 ^and 1450 cm^-1^. A fourth prominent set of peaks was observed in the putative glycogen and nucleic acid associated regions between 1000 cm^-1 ^and 1100 cm^-1^. In these latter two regions there were clear differences in peak heights between the cancer and normal median spectra although potential band overlap meant it was difficult to quantify the number of peaks or ascertain exact peak location.

**Figure 2:**
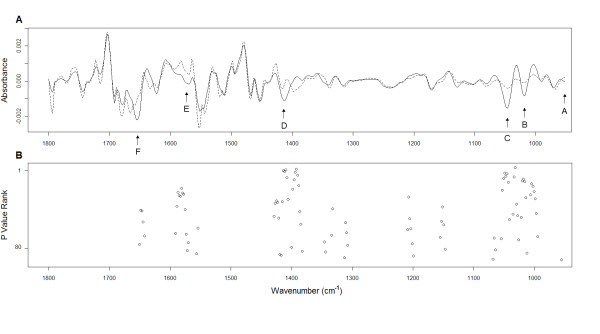
**Median second derivative spectra and significant wavenumbers**. (A) Median second derivative spectra for cancer and normal sputum. Six major significant peaks are identified (A-F) as described in Table 1. (B) Positions of 92 peaks statistically significant between cancer and normal spectra. Each peak is ranked according to p-value where the lowest p-value attained the highest rank of 1.

Second order derivative spectra were generated from the raw cancer and normal median data (Figure 1D). This approach allowed us to resolve broad, overlapping bands into individual bands thus increasing the accuracy of analysis. By applying the Mann-Whitney *U *test to each second derivative wavenumber signal, and adjusting each p-value because of multiple testing, we were able to determine a set of 92 significant wavenumbers that differentiated cancer sputum from normal. Significant wavenumbers were then ranked according to p-value and plotted against the second derivative median spectra for each group (Figure 2). This approach allowed us to quickly filter out significant wavenumbers which were actual peak centres. By analysis of the spectral alignment we determined 6 highly significant wavenumbers that could be associated with prominent and interpretable second derivative peaks in both groups (labelled as A-F in Figure 2). Table 1 summarizes each of these wavenumbers along with the proposed vibrational modes and primary molecular source.

**Table 1:** Frequency (cm^-1^) assignment, proposed vibrational mode and molecular source of 6 prominent significant wavenumbers.

Peak	Peak position from second derivative (cm^-1^)		Proposed vibrational mode	Proposed primary source
			
	normal	cancer		
A	964	966	PO_4_^= ^stretch; C-C stretch	Protein and nucleic acid
B	1024	1024	C-O stretch, C-O bend	Glycogen
C	1049	1051	C-O stretch, C-O bend	Glycogen
D*	1411	1417	COO- stretch, C-H bend	Protein
E	1577	1577	Amide II, NH bend, C-N stretch, C = N imidazole ring stretching	Protein and nucleic acid
F	1656	1654	Amide I, C = O stretch	Protein

Assignments taken from references: [22,24-26].

The small but significant band at 964 cm^-1 ^in normal sputa shifts to the 966 cm^-1 ^position in cancer sputa. This band is thought to result from vibrational modes of nucleic acids [28] and symmetrical stretching of phosphate monoesters of phosphorylated proteins [27]. Two of the most significant changes between cancer and normal sputa were seen at peaks 1024 cm^-1 ^and 1049 cm^-1 ^in the glycogen rich region. Absorbance levels were increased at these positions in all cancer cases relative to controls. Both these wavenumbers are attributed to C-O stretching and C-O bending typical of glycogen [27].

Despite removing mucus during the process of extracting cells from sputum we assumed that mucins might still be present in each sample pellet used for FTIR. The presence of mucins has been shown to cause peaks at 1040 cm^-1^, 1076 cm^-1 ^and 1120 cm^-1 ^in FTIR spectra due to C-O stretching [29]. It is possible that patients with lung cancer might simply produce more mucus leading to peak changes at these positions. Significant peaks, although less prominent, were observed between 1062 cm^-1 ^and 1066 cm^-1 ^(Figure 2B) but no peak was present at or around 1076 cm^-1 ^or 1120 cm^-1 ^in any spectrum. Similarly, no peak was apparent at 1040 cm^-1 ^however this position lies within a broad peak (Figures 1C, D) with a maxima at 1049 cm^-1^. Thus, without further analysis, it is difficult to rule out a mucin related peak being present at 1040 cm^-1^.

A peak at 1411 cm^-1^, associated with COO- stretching and C-H bending, was significantly increased in cancer spectra relative to normal. Furthermore, there was a shift in peak position from 1417 cm^-1 ^seen in normal spectra. Peaks at 1577 cm^-1 ^in the amide II region and around1656 cm^-1 ^in the amide I region were both elevated in cancer spectra relative to control. These bands characteristically reflect bending of N-H bonds and stretching of C-N bonds as well as stretching of C = O bonds [27]. In cancer spectra there was also a peak shift from 1656 cm^-1 ^to 1654 cm^-1^. Interestingly, none of these peaks were significantly different between adenocarcinoma and squamous cell carcinoma spectra

### Multivariate analysis of sputum FTIR spectra

The second derivative absorbance values at 964 cm^-1^, 1024 cm^-1^, 1411 cm^-1^, 1577 cm^-1 ^and 1656 cm^-1 ^were further subjected to multivariate analysis (MVA). The 1049 cm^-1^peak was not considered due to the potential association with mucin presence. We used two MVA methods to determine the underlying structure of how cancer and normal sputa samples grouped according to their spectra of significant wavenumbers. Application of MVA performed in this way would allow us to visualize the causal effects of significant wavenumbers on the patterns of differences existing both within and between cancer and normal sputum groups. The first method, hierarchical cluster analysis (HCA) would allow visualization of the overall grouping structure and consequently sub-groups of spectra. The second method, principal components analysis (PCA), would provide further information on how spectra group indicating which wavenumbers cause such groupings.

**Figure 3:**
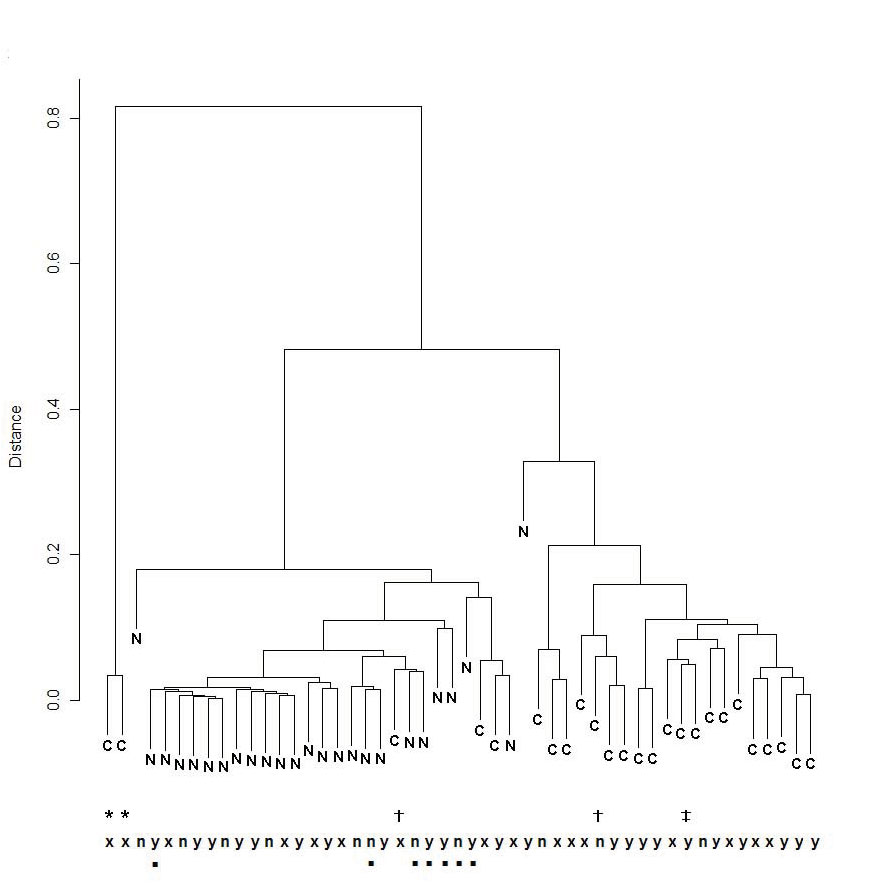
**HCA of prominent significant wavenumbers**. Dendrogram showing general and sub-clusters of lung cancer (C) and normal (N) sputum spectra produced by HCA using significant panel of wavenumbers. Supplementary information for samples are provided at the bottom of the plot: y = smoker, n = never-smoker, x = ex smoker, Previous diagnoses of other cancers are also labelled: * = breast, ‡ = larynx and bladder, † = colorectal. Normal cases who had stated that they had a cough prior to providing sputum are labelled with ■.

The HCA dendrogram for all 25 cancer and 25 normal sputum is shown in Figure 3. Two large sub-clusters are clearly visible in the dendrogram suggesting that these wavenumbers discriminate the two groups with high accuracy. Working from left to right, the first major sub-cluster contains all but two of the normal spectra. Seventeen (68.0%) normal spectra grouped tightly in a smaller sub-cluster. Interestingly, 5 out of 6 cases grouping away from this sub-cluster recorded a cough prior to providing a sputum sample. The second major cluster contained all cancer spectra as well as one normal spectrum and another that is an obvious outlier within this group. This cancer cluster had a distinct sub-cluster containing 15 (60.0%) cases. Interestingly, the four cases that had previously been diagnosed with either colon or breast cancer grouped outside this sub-cluster. Furthermore, the two cases with previous breast cancer grouped together as a pair. No sub-clustering was evident for final histology and no cluster patterns emerged for smoking status in either the cancer or normal groups. The 'tightness' of spectra within the main sub-clusters described indicates a high degree of similarity across wavenumber absorbance patterns within these groups.

**Figure 4:**
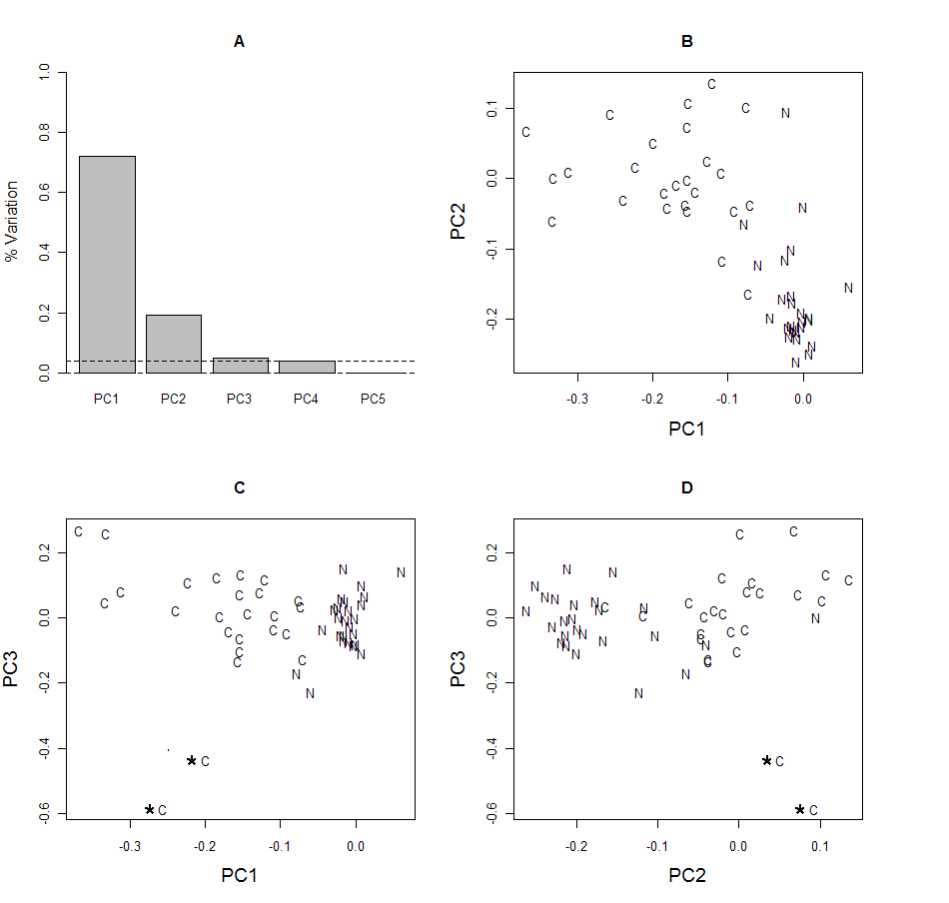
**PCA of prominent significant wavenumbers**. Plots produced after application of PCA to the panel of significant wavenumbers. (A) Scree plot showing the number of components to retain explaining at least 5% of the variation. The first 3 components explain 95% variance. Scatterplots of the loadings of each cancer (C) and normal (N) sputum spectrum on: (B) components 1 (PC1) and 2 (PC2); (C) components 1 and 3 (PC3); (D) components 2 and 3.

PCA returned three components that collectively explained 96.1% of the variation within the 5 wavenumbers (where each component comprised at least 5% variation). The scree plot in Figure 4A shows that these three components (PC1, PC2 and PC3) explained 71.9%, 19.2% and 5.0% of the variation each. A scatterplot of the correlation of spectra on PC1 and PC2 (Figure 4B) reveals how these components explain variation existing between cancer and normal cases. Cancer spectra had a higher negative correlation on PC1 compared to normal spectra. An assessment of wavenumber loadings on PC1 revealed that this was primarily due to high negative and positive loadings of 1024 cm^-1 ^and 1411 cm^-1 ^respectively on PC1. With the exception of an outlier, all normal spectra had a negative correlation on PC2 and this component had a high positive loading of 1656 cm^-1 ^and high negative loading of 1577 cm^-1^. The two cases previously diagnosed with breast cancer are separated from all other spectra due to a high negative correlation on PC3. Again, this can be explained by a high negative loading of 1656 cm^-1 ^on PC3.

## Discussion

This study is the first (to our knowledge) to generate FTIR spectra from sputum and derive chemical fingerprints for the purpose of diagnosing lung cancer.

With the knowledge that FTIR yielded excellent reproducibility for sample spectra, our primary objective in this study was to determine which wavenumbers were significantly different between sputum of cancer and normal controls. Prominent significant wavenumbers could then be used to explore the structure of patterns of similarities within and between both cohorts using MVA techniques. The data analysis strategy we employed was robust and took into consideration the data distribution at each wavenumber. We have found that many FTIR studies apply parametric tests to wavenumber data with no evidence of data distribution yet we found that data at all wavenumbers in this study did not follow a normal distribution. Interestingly, Whiteman *et al*. [22] also made the same observation in their study on FTIR spectra in sputum of COPD patients.

The six peaks described in Table 1 all arose due to an increase in absorbance at that spectral position in cancer relative to normal controls often with a noticeable position shift. A rise in absorbance at a wavenumber in one sample relative to another can be due to different reasons including an increase in the frequency of a bond vibration mode [27]. It should also be noted that the non-uniform distribution and degree of compaction of molecules within cells can also have a non-linear affect on absorbance level as considered for chromatin within dividing and non-dividing cells [30]. Stronger intermolecular interactions at bonds, such as C-O in carbohydrates or COO- in proteins, result in higher absorbance levelsIt is possible that an increase in absorbance levels at 1656 cm^-1 ^and 1577 cm^-1 ^is due to an increase in protein levels between cancer and normal cells in sputum [27].

Band shifts and significant differences in absorbance intensity at 1024 cm^-1 ^and 1049 cm^-1 ^may signify an increase in cancer sputum of levels in glycogen. A previous study by [18] using FTIR suggested that a glycogen band at 1045 cm^-1 ^was increased in lung tumours (squamous cell carcinoma and adenocarcinoma) relative to normal tissue. A subsequent study using FTIR microscopy confirmed increased levels of glycogen in lung tumour cells [19]. FTIR showed glycogen levels were also increased in lung tumour cells of pleural fluid due to an increase in absorbance at 1030 cm^-1 ^in the glycogen rich region [24]. An increase or decrease in glycogen levels varies according to cancer type, for example levels are higher in colon tumour tissue relative to normal but a reduction is seen in tumours of the cervix and liver [18]. Indeed, the significant increase in absorbance levels at 1024 cm^-1 ^and 1049 cm^-1 ^in lung cancer sputa observed in our study contrast the decreased levels for these wavenumbers in tumours of the cervix compared to normal tissue [27]. Importantly, if differences in absorbance at 1024 cm^-1 ^and 1049 cm^-1 ^wavenumbers between lung cancer and normal sputa cells represent glycogen levels then our results confirm previous studies and suggest that increased glycogen levels, via detection by FTIR, could prove to be a powerful diagnostic factor for lung cancer.

The significant band shift at 964 cm^-1 ^has been associated with symmetrical stretching in bonds of phosphorylated proteins but has also been associated with cancer related structural change in nucleic acids [28]. The band increase (and shift) at 1411 cm^-1 ^in cancer sputum spectra might be an indicator of a change in nucleic acid level [31] alternatively, this change could also suggest COO- stretching and C-H bending due to proteins. Our results suggest that further work should explore the contribution different molecules that differentiate lung cancer from normal sputum spectra so accurate assignments of the causation of absorbance changes can be made.

An important consideration when generating FTIR spectra from biospecimens such as sputum is that cells will often be derived from mixed tissue types which can lead to spurious results [32]. In this study, we ensured that each sputum sample was assessed by a pathologist for sufficient presence of bronchial epithelial cells. Indeed, validation of our work could involve analysis of cell pellets by FTIR microspectroscopy to generate spectra for pre-identified normal bronchial and tumour cells.

Using 5 significant wavenumbers we were able to visualize the underlying structure of how all sputum samples grouped according to patterns of differences in absorbance levels. HCA and PCA in combination allowed visualization and interpretation of spectral sub-groups. The observation from the HCA dendrogram (Figure 3) that no sub-groupings emerged due to histological type suggests that the 5 significant wavenumbers are not type specific. Perhaps a much larger set of samples of NSCLC subtypes and especially small cell carcinoma cases may reveal FTIR spectral differences according to tumour sub-type.

PCA gave further insight into the causal relationship between groupings of spectra and individual significant wavenumbers with 3 components explaining 96.1% of the variation. The first two components (PC1 and PC2) show that cancer spectra clearly separate from normal spectra according to the loadings on the protein, glycogen and DNA associated wavenumbers 1577 cm^-1^, 1024 cm^-1^and 1411 cm^-1^. These wavenumbers are thus important potential diagnostic markers for lung cancer. PC3 was highly associated with the two spectra for patients who had previously been diagnosed with invasive ductal carcinoma of the breast. This result is interesting as both cases had a final histology of NSCLC yet, PCA reveals that both spectra have a high similarity to each other but are separated from other lung cancer spectra. Although data is extremely limited one might hypothesize that FTIR has the potential to further discriminate metastatic tumours where the primary arose in the breast.

Throughout the analysis we were mindful of confounding variables that might lead to misinterpretation of differences between cancer and normal sputum spectra. It is suggested that inflammation plays a key role in the pathogenesis of lung cancer [33]. From the patient medical histories recorded we noted conditions that could contribute to inflammation in the bronchial tubes. For example, a number of cancer cases had also been diagnosed with COPD or asthma according to standard criteria. Furthermore, the control group also included cases with COPD and asthma. However, an inspection of the grouping patterns of HCA and PCA did not reveal any similarities either within group or between groups due to the presence of these conditions. It is interesting to note however that spectra of nearly all the normal cases who had presented with a cough (prior to sputum acquisition) were more dissimilar to the large sub-cluster of normal spectra in the HCA dendrogram. We were not however able to find any association of wavenumbers with these few cases using PCA.

The spectra of cancer and COPD from sputum can be further compared in detail. Whiteman *et al*. [22] compared the FTIR spectral profiles from sputum of 15 COPD patients and 15 healthy volunteers. That study yielded reproducible spectra from sputum with no significant difference between patterns in smokers and non-smokers, factors that are mirrored in our study. The key findings of the COPD study were that major spectral changes between groups were observed as peak shifts in the regions of 1559 cm^-1^, 1077 cm^-1 ^and 1458 cm^-1^. Thus, in sputum, the significant pattern of change in FTIR spectra of COPD patients is different to that seen in cancer patients. Whiteman *et al*. conclude from their study that the main contributor shaping the heterogeneous FTIR spectrum in COPD patient sputa is *in vivo *airway inflammation. If this is the case then airway inflammation is not a major contributor to the lung cancer sputum spectrum strengthening the argument that the molecular changes observed are cancer-specific.

It was also important to ensure that absorbance at key wavenumbers were not changed in cancer sputa simply due to differing levels of mucus despite the removal process. Absorbance levels of key mucus related peaks at 1076 cm^-1 ^and 1120 cm^-1 ^were either very low or non-existent. Absorbance levels of another mucus related peak at 1040 cm^-1 ^were more difficult to establish as this wavenumber was situated in the shoulder of the glycogen related 1049 cm^-1 ^peak. Removal of the 1049 cm^-1 ^wavenumber during analysis ensured that differences between cancer and normal sputa were not influenced by presence of mucus.

Although the HCA dendrogram demonstrates a clear separation between the major cancer and normal clusters two normal spectra did group with cancer spectra. Thus, an important question arising from this study is: what are the potential levels of specificity and, more importantly, sensitivity when using the panel of wavenumbers to discriminate cancer from normal sputum? An exact figure should not be estimated from just 50 cases but the grouping patterns observed using MVA suggests that sensitivity and specificity would be at least greater than 80% which compares more than favourably with existing methods of lung cancer detection.

## Conclusions

In conclusion, we report the preliminary application of FTIR to determine biochemical changes in sputum between lung cancer and normal cases. Our results suggest that FTIR applied to sputum might have a high sensitivity and specificity in diagnosing the disease using a small panel of significant wavenumbers. The continuous collection of sputum within the Medlung project will allow us to generate predictive models for lung cancer on much larger datasets. The cases used in this study were recruited mainly at bronchoscopy so tended to have more centrally localised tumours. Thus, we are currently investigating the ability of FTIR to detect peripheral lung tumours using sputum and are encouraged by the fact that FTIR was able to detect cancer in 48% of cases where no tumour was visible during bronchoscopy. If biochemical changes in sputum can also be detected by FTIR in the early stages of lung cancer, then the technology might prove to be a non-invasive, cost-effective, high-throughput method for eventual screening. With this goal in mind, we are also performing a longitudinal study to determine whether the panel of infrared wavenumbers can also discriminate patients deemed at high-risk for lung cancer.

## Competing interests

The authors declare that they have no competing interests.

## Authors' contributions

PDL conceived the study, performed data analysis and participated in its design and supervision. LUM participated in study design and coordinated FTIR. KEL, RG and PK coordinated tissue and data collection and provided clinical input into the study. SB, AJL and JW performed FTIR. ARG provided intellectual input into the study and helped draft the manuscript. All authors read and approved the final manuscript.

## Pre-publication history

The pre-publication history for this paper can be accessed here:

http://www.biomedcentral.com/1471-2407/10/640/prepub
